# Bioactive Components and Potential Mechanism Prediction of Kui Jie Kang against Ulcerative Colitis via Systematic Pharmacology and UPLC-QE-MS Analysis

**DOI:** 10.1155/2022/9122315

**Published:** 2022-06-21

**Authors:** Jinbiao He, Chunping Wan, Xiaosi Li, Zishu Zhang, Yu Yang, Huaning Wang, Yan Qi

**Affiliations:** ^1^College of Pharmaceutical Science, College of Clinical Medicine and School of Basic Medicine, Yunnan University of Chinese Medicine, Kunming, Yunnan 650500, China; ^2^Traditional Chinese Medicine Department, Yunnan Health Rehabilitation Centre, Kunming, Yunnan 650307, China

## Abstract

Kui Jie Kang (KJK)—a traditional Chinese medicine—has demonstrated clinical therapeutic efficacy against ulcerative colitis (UC). However, the active compounds and their underlying mechanisms have not yet been fully characterized. Therefore, the current study sought to identify the volatile compounds in KJK responsible for eliciting the therapeutic effect against UC, while also analyzing key targets and potential mechanisms. To this end, systematic network pharmacology analysis was employed to obtain UC targets by using GeneCards, DisGeNET, OMIM, among others. A total of 145 candidate ingredients, 412 potential targets of KJK (12 herbs), and 1605 UC targets were identified. Of these KJK and UC targets, 205 intersected and further identified AKT1, JUN, MAPK, ESR, and TNF as the core targets and the PI3K/AKT signaling pathway as the top enriched pathway. Moreover, molecular docking and ultra-performance liquid chromatography Q Exactive-mass spectrometry analysis identified quercetin, kaempferol, luteolin, wogonin, and nobiletin as the core effective compounds of KJK. *In vivo* murine studies revealed that KJK exposure increases the body weight and colon length, while reducing colonic epithelial injury, and the expression of inflammatory factors in colitis tissues such as TNF-*α*, IL-6, and IL-1*β*. Furthermore, KJK treatment downregulates the expression of *pi3k* and *akt* genes, as well as p-PI3K/PI3K and p-AKT/AKT proteins. Collectively, these findings describe the therapeutic effects and mechanisms of KJK in UC and highlight KJK as a potentially valuable therapeutic option for UC via modulation of the PI3K/AKT signaling pathway, thus providing a theoretical reference for the broader application of KJK in the clinical management of UC.

## 1. Introduction

Inflammatory bowel disease is a chronic, recurrent, and autoimmune disease that primarily occurs in the colon and bowel and includes Crohn's disease and ulcerative colitis (UC) [1, 2]. UC is a common diffuse, nonspecific, and recurrent inflammatory disease of the rectum and colon mucosa and submucosa with high rates of relapse and canceration [3]. The incidence of UC has recently increased [4] with over 6.8 million cases having been reported worldwide [5]. Moreover, the age of onset tends to be 20–40 years old and can also be seen in children or the elderly [3]. It can be characterized by periods of remission followed by repeated flare-ups [3]. Its symptoms include belly aches, bloody diarrhea, proctorrhagia, and fatigue in addition to symptoms outside of the intestine, such as peripheral arthritis, noninfective conjunctivitis, and weight loss, which affect the quality of life and economic status of patients [6]. Meanwhile, during the occurrence and development of UC, genetic susceptibility, destruction of the epithelial barrier, and abnormal intestinal flora may lead to dysregulation of the immune response and abnormal inflammatory signals [7]. However, the precise etiology of UC is complex and not yet fully defined. Current nonsurgical treatment strategies, including glucocorticoids, are limited by high recurrence rates and adverse side effects [8]. Therefore, the development of effective treatments for UC with fewer side effects is an important goal in medical research.

Kui Jie Kang (KJK) comprise *Poria cocos* (Fu Ling), *Paeoniae radix* (Bai Shao), *Citri Reticulatae Pericarpium* (Chen Pi), *Codonopsis pilosula* (Dang Shen), *Atractylodes macrocephala* (Bai Zhu), *Saposhnikovia divaricata* (Fang Feng), *Bletilla striata* (Bai Ji), *Sanguisorba officinalis* (Di Yu), *Dioscorea rhizoma* (Shan Yao), *Panax notoginseng* (San Qi), *Patrinia villosa* (Bai Jiang Cao), and *Platycodon grandiflorus* (Jie Geng). It is a medication, based on an ancient Chinese “Tongxie” prescription, that has demonstrated clinical therapeutic efficacy. We have previously explored the preventive and therapeutic effects of this prescription in UC from various perspectives and found that KJK can downregulate NF-*κ*B expression, inhibit the intestinal inflammatory response, restore intestinal mucosal barrier function, reduce the oxidative stress response, inhibit NLRP3 inflammasome activation, and regulate intestinal flora imbalance in a mouse model of UC. However, the effective components, key targets, and additional regulatory pathways for intervention remain unclear.

Multi-pharmaceutical research is the basic strategy for the clinical application of traditional Chinese medicine (TCM). Although it is generally believed that the effective ingredients of TCMs are chemicals in drugs or metabolites, TCMs usually contain thousands of compounds, whose components may be altered by environmental factors or preparation methods [9]. In addition, prototypes used by humans or animals may undergo a comprehensive biological reaction to produce a variety of metabolites. Therefore, screening all drug-related compounds from biological matrices represents a significant challenge. In this study, we determined the effective ingredients, therapeutic targets, and mechanism of action of KJK. The potential therapeutic targets were determined using network pharmacological analysis, while the herbal chemical effective ingredients and underlying pharmacological mechanisms were identified using Q Exactive MS/MS (UPLC-QE-MS) and an *in vivo* UC mouse model. These results provide new insights regarding the mechanism by which KJK contributes to the treatment of UC and provides a reference for its broader clinical application.

## 2. Materials and Methods

### 2.1. Chemicals and Reagents

Chinese herbs of KJK were purchased from the Department of Pharmacy, the First Affiliated Hospital of Yunnan University of Traditional Chinese Medicine (Yunnan, China). Mesalazine enteric coated tablets (national medicine permission number: H19980148) were purchased from Sunflower Pharmaceutical Group Jiamusi Luling Pharmaceutical Co., Ltd. (Heilongjiang, China). The SDS-PAGE Gel Matching Kit (cat. no. P0012A) and BCA Protein Determination Kit (cat. no. P0012) were purchased from Beyotime Biotech Inc. (Shanghai, China). Additionally, 4% paraformaldehyde was obtained from Sinopharm (Beijing, China). Primers were designed by Generay Biotech Co. Ltd. (Shanghai, China). A Total RNA Extraction Kit (cat. no. DP419) was obtained from the Tiangen Biochemical Department, Technology Co., Ltd. (Beijing, China). TaKaRa PrimeScript RT Master Mix (cat. no. RR036A) and TaKaRa TB Green Premix Ex Taq II (cat. no. RR820A) were purchased from Bao Biological Engineering (Dalian) Co. (Dalian, China). The anti-MPO antibody (ab9535) was purchased from Abcam, Inc. (Abbott Park, IL, USA), and PI3K rabbit polyclonal antibody (cat. no. 4257S), p-PI3K rabbit polyclonal antibody (cat. no. D0615), AKT rabbit polyclonal antibody (cat. no. 4685S), and p-AKT rabbit polyclonal antibody (cat. no. 4060S) were purchased from Cell Signaling Technology (Danvers, MA, USA). IRDye®680RD Goat anti-Rabbit IgG Secondary Antibody (cat. no. 926–68071) was purchased from LI-COR Biotechnology (Nebraska, USA). The GAPDH rabbit polyclonal antibody (cat. no. 10494-1-AP) was purchased from Proteintech (Chicago, IL, USA). DSS was purchased from MP Biomedicals (Irvine, CA, USA). IL-1*β* (cat. no. MB-2906A), IL-6 (cat. no. MB-2899A), TNF-*α* (cat. no. MB-2868A), and enzyme-linked immunosorbent kits were purchased from Jiangsu Enzyme Labeling Biotechnology Co. (Jiangsu, China).

### 2.2. Analysis of the Correlation between Kui Jie Kang and Ulcerative Colitis

Through TCM Network Pharmacology Analysis System (TCMNPAS, https://54.223.75.62:3838/), correlations between KJK and its components and the molecular basis of UC were evaluated. The specific operations were as follows: input UC (ULC004) and KJK (composition: *Atractylodes macrocephala*, *Poria cocos*, *Paeonia alba*, tangerine peel, Fangfeng, *Platycodon grandiflorum*, *Panax notoginseng*, *Sophora japonica*, *Sanguisorba officinalis*, and licorice), select hit, TCMID, stitch, and TCMSP databases, parameter property index QED set to 0.2, drug relevance threshold set to 400, compound target significance set to *P* < 0.05 [10]. Gene ontology-cellular component (GO-CC), gene ontology-biological process (GO-BP), gene ontology-molecular function (GO-MF), and Kyoto Encyclopedia of Genes and Genomes (KEGG) UC enrichment analyses were performed. Default settings were used for the remaining parameters.

### 2.3. Network Pharmacology Analysis

Using TCMSP (https://tcmspw.com/index.php), a platform for the systematic pharmacological analysis of TCM, searches were performed for KJK active components and targets. Each component of KJK was used as an input. Oral availability (OB) was set to ≥30%, and drug similarity (DL) was set to ≥0.18 [11]. The unverified active ingredient targets were then obtained from the SwissTarget Prediction (https://www.swisstargetprediction.ch/) database. Using the PubChem database (https://www.lilab-ecust.cn/pharmmapper/), the active ingredients were imported in mol2 format, setting the species to human; 10 targets with the highest fit score were obtained by default. The UniProt database (https://www.uniprot.org/) defines the species as human, corrects the target corresponding to each active ingredient, and converts it into the corresponding gene name for further mechanistic research. With human genes as the background, “ulcerative colitis” was used as the search term for the identification of related genes in GeneCards (https://www.genecards.org/), DisGeNET (https://www.disgenet.org/), OMIM (https://omim.org/), and TTD (http://db.idrblab.net/ttd/). An information table including disease targets was exported, and redundant data were deleted.

Using Venny 2.1 (https://bioinfogp.cnb.csic.es/tools/venny/), a Venn diagram of the KJK targets and UC targets was generated. The active components of KJK and UC-related genes were introduced to the STRING database (https://string-db.org/) to evaluate core genes by the construction of a protein-protein interaction network, with “Homo sapiens” as the organism, a minimum required interaction score of 0.70 (high confidence), and deletion of highly disconnected nodes. Thereafter, a molecule network analysis was conducted using Cytoscape v3.8.2 and the CytoNCA plug-in. Pathway annotation of the core targets was performed using DAVID (https://david.ncifcrf.gov/). GO-BP, GO-CC, and GO-MF enrichment, and KEGG enrichment analyses were carried out (*P* ≤ 0.0001, count >5). The “drug-target-pathway” table was imported into Cytoscape to generate network pharmacology figures.

### 2.4. Molecular Docking Analysis

AutoDockTools 1.5.6 was used to dock the KJK core active ingredients and the target proteins based on the UC target pathway. Autogrid calculations were performed, selecting AutoDock Vina as the docking algorithm, and AutoDock was used for molecular docking. A binding energy of less than −5 kcal/mol was selected as the reference, and the binding energy results were visualized using PyMOL 2.4.0. Moreover, hydrophobic force analysis was conducted via the website analysis (https://plip-tool.biotec.tu-dresden.de/).

### 2.5. Preparation of Kui Jie Kang

Purified water equivalent to 10 times the weight of KJK was added, boiled for 1 h, and filtered. An eight-fold volume of water was added to the filter residue, decocted for 40 min, and filtered. A six-fold volume of water was added to the filter residue and decocted for 30 min. The filtrates were combined three times, filtered, concentrated in the rotary evaporator to prepare the KJK flow extract, and stored in a refrigerator at −20°C until use.

### 2.6. UPLC-QE-MS Analysis

The sample (100 mg) was supplemented with 500 mg/*μ*L extract (methanol: water = 4 : 1, internal standard concentration: 10 *μ*g/mL), vortexed for 30 s, homogenized at 45 Hz for 4 min, and added to an ultrasonic (Shenzhen Fang'ao Microelectronics Co., YM-080S, Shenzhen, China) ice water bath for 1 h. After standing at −40°C for 1 h, samples were centrifuged at 4°C and 12004 × *g* for 15 min. The upper layer was carefully removed and subjected to 0.22 *μ*M microporous membrane filtration. Samples were stored at −80°C until use. LC-MS/MS was performed using a Agilent ultra-high-performance liquid chromatography 1290 UPLC system with a Waters UPLC BEH C18 column (1.7 *μ*m, 2.1 × 100 mm). The flow rate was set to 0.4 mL/min, and the sample injection volume was set to 5 *μ*L. The mobile phases consisted of 0.1% formic acid in water (A) and 0.1% formic acid in acetonitrile (B). The multi-step linear elution gradient program was carried out, and MS and MS/MS data were obtained as previously described [12].

### 2.7. Animal Experiment

All animal protocols were reviewed and approved the Intramural Committee on the Ethical and Humane Treatment of Experimental Animals of Yunnan Traditional Chinese Medicine Hospital/Medical Ethics Committee of the First Affiliated Hospital of Yunnan University of Traditional Chinese Medicine, and were in accordance with the NIH Guidelines for the Care and Use of Laboratory Animals (Application No: SD2021-001).

C57BL/6 female mice (18 ± 2 g) were purchased from Dashuo Biotech Co. (SLAC, Hunan, China). All mice were housed under standard conditions (12-h dark-light cycle; 25 ± 2°C; humidity 50–75%) with free access to food and water. Mice were acclimated by placing them on a diet administered *ad libitum* for one week. They were then randomly divided into six groups, with 10 mice per group, as follows: normal diet control group (CON), 2.5% DSS group (MOD), 2.5% DSS + mesalazine group (MC), 2.5% DSS+10.08 mg/kg KJK group (KJK-H), 5.04 mg/kg KJK group (KJK-M), 2.5% DSS + 2.52 mg/kg KJK (KJK-L). Excluding the CON group, all groups had *ad libitum* access to 2.5% DSS (MW: 36000–50000 Da) solution for 7 days, and fresh DSS solution was added each day. Next, the CON and MOD groups were given normal saline, the MC group was administered mesalazine, and the remaining groups were administered corresponding doses of KJK for one week. The weight, diarrhea, and rectal bleeding of mice were recorded every 2 days, and the disease activity index (DAI) score was calculated as described in Table 1. At the end of the study, all mice were fasted for 12 h, and anesthesia was induced via intraperitoneal injection of 1% sodium pentobarbital at a dose of 0.04 ml/10 g mice. Blood was collected from the orbital cavity and centrifuged [13]. Samples of colon tissue were collected and stored in liquid nitrogen, while feces were stored at −80°C until use. In addition, colonic tissue of mice was taken and placed in 4% paraformaldehyde solution for further histopathological observation.

### 2.8. Histopathological Observation of the Colon

The colon tissue was fixed with 4% paraformaldehyde for 24 hours and then sectioned by dehydration, embedded in paraffin, and cut into 5–7 *μ*m sections, which were stained with hematoxylin-eosin (H & E) and examined under a light microscope (Thermo, Waltham, MA, USA) at 200× magnification. Five animals in each group were observed under the microscope in four fields of view each for changes in colonic tissue.

### 2.9. Enzyme-Linked Immunosorbent Assay

Precisely weigh 80 mg of mice colon tissue into 720 *μ*l of phosphate-buffered saline buffer (pH 7.4) and homogenize. The tissue was centrifuged at 12004 × *g* for 15 min at 4°C. The supernatant was then collected, and BCA quantification was performed. IL-1*β*, IL-6, and TNF-*α* were quantified by ELISA kits according to the manufacturer's protocol.

### 2.10. RNA Extraction and Real-Time Quantitative PCR

Total RNA was prepared from the colon using TRIzol reagent according to the manufacturer's instructions. Total RNA (1 *μ*g) was equalized and converted to cDNA using the HiScript II Reverse Transcriptase Kit. The reaction system was as follows: PrimeScript RT Master Mix 4 *μ*L, 1000 ng of total RNA, and ddH_2_O to 20 *μ*L volume. The reaction conditions were as follows: 37°C for 15 min and 85°C for 5 s. Gene expression was measured by RT- qPCR (Roche, Basel, Switzerland) using SYBR green. A total of 1 *μ*L of cDNA template was used for PCR amplification using the following primers: *pi3k* F, 5′-TCTACCCAGTGTCCAAATACCA-3´; R, 5′-AAATGCTTCGATAGCCGTTCT-3′; *akt* F, 5′-CGACCGCCTCTGCTTTGT-3′; R, 5′-AAGTCCGTTATCTTGATGTGCC-3′. The total reaction volume was 20 *μ*L, including SYBR Premix Ex Taq II (10 *μ*L), Primer F (0.3 *μ*L), Primer R (0.3 *μ*L), cDNA (1 *μ*L), ddH_2_O (8.4 *μ*L). The PCR conditions were as follows: pre-denaturation at 95°C for 30 s, denaturation at 95°C for 5 s, annealing and extension for 20 s at 62°C, and amplification for 40 cycles. The ratio of target products to the internal reference *GAPDH* gray value (CT value) was calculated to determine the relative mRNA expression levels of *pik3* and *akt*.

### 2.11. Western Blotting

Colon tissue samples were homogenized in radioimmunoprecipitation assay buffer until they were completely lysed, and the supernatants were collected by centrifugation. The total protein concentration was determined using the Bicinchoninic Acid (BCA) Protein Assay Kit. 50ug protein was separated by electrophoresis and transferred onto polyvinylidene difluoride (PVDF) membranes. The membranes were blocked with 5% skim milk for 1 h at room temperature and then incubated with primary antibodies against p-PI3K/PI3K and p-AKT/AKT (dilute with primary antibody diluent in a dilution ratio of 1 : 1000) overnight at 4°C. The membranes were then washed and incubated with a horseradish peroxidase-labeled secondary antibody for 1 h at room temperature, and the signal was detected using an enhanced fluorescent luminescent substrate. Immunoreactive bands were quantified using ImageJ, and the density ratio of the target protein to GAPDH was used as the relative content of the target protein for statistical analyses.

### 2.12. Data Analysis

Statistical analyses were performed using SPSS version 21.0 (IBM, Armonk, NY, USA). Measurement data are expressed as mean ± SEM. One-way analysis of variance (ANOVA) was conducted for group comparisons; differences were considered statistically significant at *P* < 0.05.

## 3. Results

### 3.1. Associations between Kui Jie Kang Components and Ulcerative Colitis

**Figure 1:**
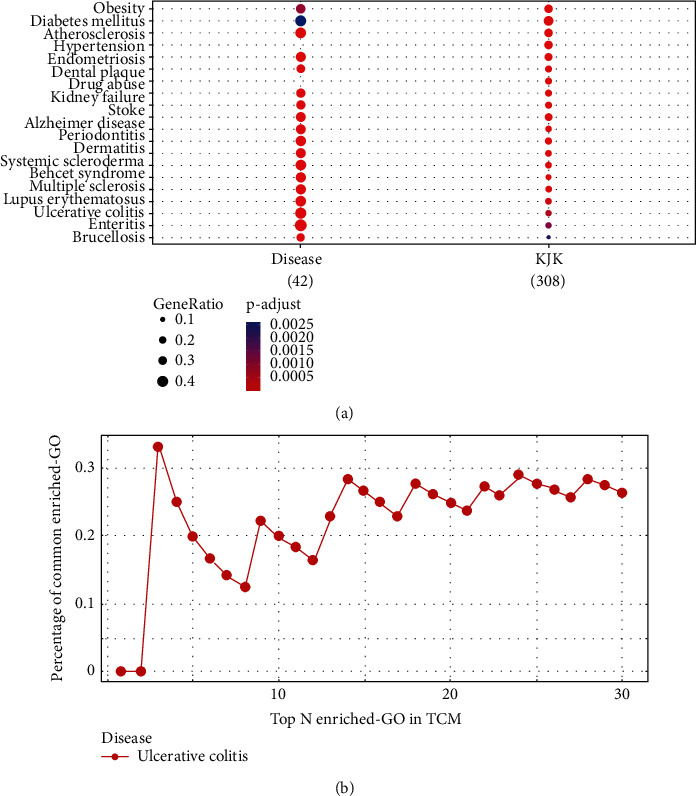
Correlation analysis between Kui Jie Kang (KJK) and ulcerative colitis (UC). (a) The KJK and UC correlation was significant (*P*=0.000191). (b) Top 30 enriched GO terms in traditional Chinese medicine (TCM).

We used the TCMNPAS database to evaluate the genes co-associated with various diseases and drugs to establish the therapeutic value of KJK in UC (Figure 1(a)). This analysis of the TCMNPAS database revealed a significant overlap between target genes of the KJK components and the genes related to UC (*P*=0.000191). In addition, the similarity of co-associated GO terms between KJK and UC was 0.74; the AUC for the top 30 co-associated GO terms between KJK and UC was 6.61 (Figure 1(b)). To a certain extent, these findings support the clinical value of KJK in UC.

### 3.2. Network Pharmacology Analysis

**Figure 2:**
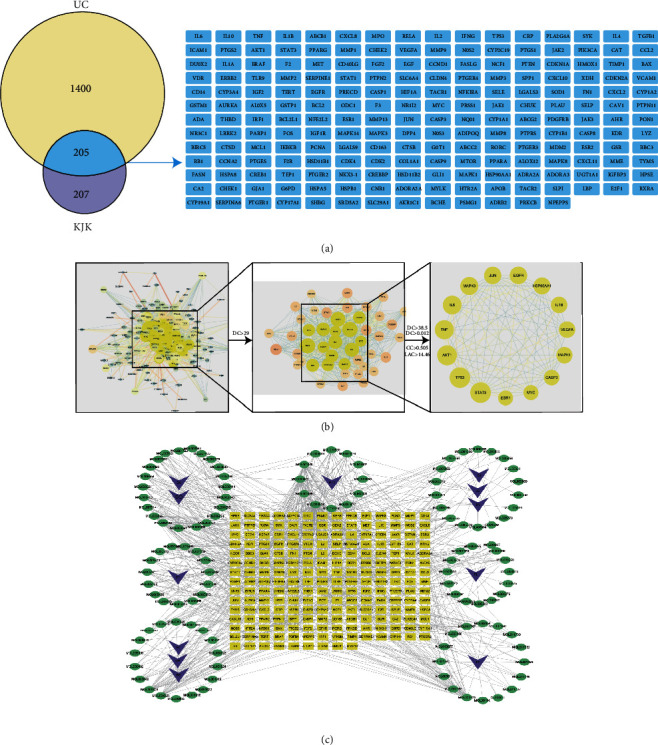
Network pharmacology prediction for KJK treatment of UC. (a) Venn diagram of KJK in the treatment of UC. (b) The protein-protein interaction network. (c) KJK ingredient-UC target interaction network. Green circle: active ingredient; orange square: gene target; purple arrow head: the higher the node degree value, the larger the node diameter and the darker the color. Moreover, the stronger the interaction between nodes, the thicker the edge and the darker the color. TCM. DS: *Codonopsis pilosula*, BZ: *Atractylodes macrocephala*, FL: *Poria cocos*, BS: *Paeoniae radix*, CP: *Citri Reticulatae Pericarpium*, FF: *Saposhnikovia divaricata*, JG: *Platycodon grandiflorum*, BJ: *Bletilla striata*, SY: *Dioscorea rhizoma*, SQ: *Panax notoginseng*, BJC: *Patrinia villosa*, DY: *Sanguisorba officinalis*.

In the TCMSP database, 145 active ingredients in KJK were identified: *Codonopsis pilosula* had 21 active ingredients, *Atractylodes macrocephala* had 7, *Poria cocos* had 15, *Paeoniae radix had* 13, *Citri Reticulatae Pericarpium* had 5, *Saposhnikovia divaricata* had 18, *Platycodon grandiflorum* had 7, *Bletilla striata* had 9, *Dioscorea rhizoma* had 16, *Panax notoginseng* had 8, *Patrinia villosa* had 13, and *Sanguisorba officinalis* had 13 active ingredients. In total, 412 KJK targets were obtained after target de-duplication using the SwissTargetPrediction database. A total of 1605 UC-related targets were obtained from the GeneCards, OMIM, and DrugBank databases, with 205 of the UC-related and KJK targets found to intersect (Figure 2(a)).

The STRING database and Cytoscape 3.8.2 were used to construct a protein-protein interaction (PPI) network of targets (Figure 2(b)). According to a topological analysis, the network had 192 nodes and 1829 edges. In total, 46 nodes and 576 edges were obtained by applying a screening threshold of twice the median degree value of 29. Finally, 15 targets, meeting these parameters, were screened with a median degree value of 38.5, betweenness centrality (BC) of 0.012281205, local average connectivity (LAC) of 16.46029412, and closeness centrality (CC) of 0.50529101 (Table 2). These were identified as candidate targets by which KJK contributes to the treatment of UC. Based on a network diagram of KJK active ingredients and UC targets constructed using Cytoscape 3.8.2, we speculated that quercetin, kaempferol, luteolin, wogonin, and nobiletin may be the key components by which KJK exerts its beneficial effects against UC (Figure 2(c)).

### 3.3. GO and KEGG Pathway Enrichment Analysis

**Figure 3:**
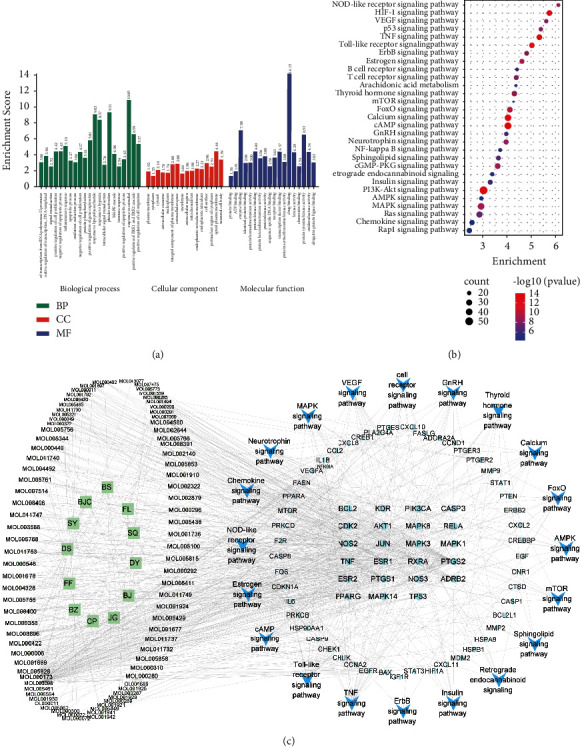
Analysis of UC treatment with KJK using gene ontology (GO) and pathway enrichment analyses. (a) GO enrichment analysis for the key targets. (b) Kyoto Encyclopedia of Genes and Genomes (KEGG) pathway enrichment analysis of the key targets. (c) The KJK active ingredient-UC target pathway network. Green circle: target gene; green diamond: active ingredient; green square: TCM; blue arrow head: pathways.

A total of 205 intersecting target genes were evaluated by GO and KEGG enrichment analyses using the DAVID database. The 15 most significant terms in the GO-BP, GO-MF, and GO-CC categories at *P* < 0.0001 with a count >5 are displayed in Figure 3(a). The BP terms primarily included positive regulation of transcription from RNA polymerase II promoter, signal transduction, positive regulation of cell proliferation, negative regulation of apoptotic process, positive regulation of transcription, DNA-templated, and inflammatory response. The CC terms mainly included plasma membrane, cytoplasm, cytosol, extracellular exosome, and nucleoplasm. The MF terms primarily included protein binding, ATP binding, enzyme binding, identical protein binding, and protein homodimerization activity.

A total of 127 signaling pathways were identified via KEGG enrichment analysis. According to a significance threshold of *P* < 0.0001 and count >5, 29 disease-related pathways were screened (Figure 3(b)). The pathways with the top five highest gene counts were the PI3K-AKT (*n* = 55), cAMP (*n* = 42), MAPK (*n* = 39), calcium (*n* = 38), and Ras (*n* = 34) signaling pathways. Moreover, to elucidate the relationships among ingredients, targets, and pathways, 29 pathways identified in the KEGG enrichment analysis and intersecting genes were used to construct a target pathway network using Cytoscape 3.8.2, resulting in a “KJK active ingredient-UC target pathway” interaction network (Figure 3(c)). The pathways by which KJK exerts therapeutic effects in UC were primarily related to inflammation, immunity, metabolism, and signal transduction. Moreover, considering that the PI3K-AKT signaling pathway included the largest number of genes, it may represent the most relevant KJK active ingredient-UC target pathway.

### 3.4. Molecular Docking of the Main Active Ingredients of Kui Jie Kang and Core Proteins

By combining the results of the KJK active ingredient-UC target network, PPI network, KJK active ingredient-UC target pathway network, and GO and KEGG pathway enrichment analyses, we identified five key genes, namely, *Akt1*, *Jun*, *Mapk1*, *Esr1*, and *Tnf*. We acquired the 3D structures of the AKT1, JUN, MAPK1, ESR1, and TNF protein receptors from the PDB. Moreover, quercetin, kaempferol, luteolin, wogonin, and nobiletin were found to be ligands of AKT1 (PDB ID: 6HHF), JUN (PDB ID: 3U86), MAPK1 (PDB ID: 6G54), ESR1 (PDB ID: 5GTR), and TNF (PDB ID: 1FT4) protein receptors. The 2D structure of the small molecule compound was obtained using the PubChem database and was converted to a 3D structure using ChemOffice. The water molecules and ligands of these protein receptors were removed using PyMOL 2.4.0. We visualized the 3D binding of quercetin, kaempferol, luteolin, wogonin, and nobiletin to AKT1, JUN, MAPK1, ESR1, and TNF protein receptors using AutoDockTools and AutoDock Vina. A lower binding energy indicated a more stable structure (Table 3).

**Figure 4:**
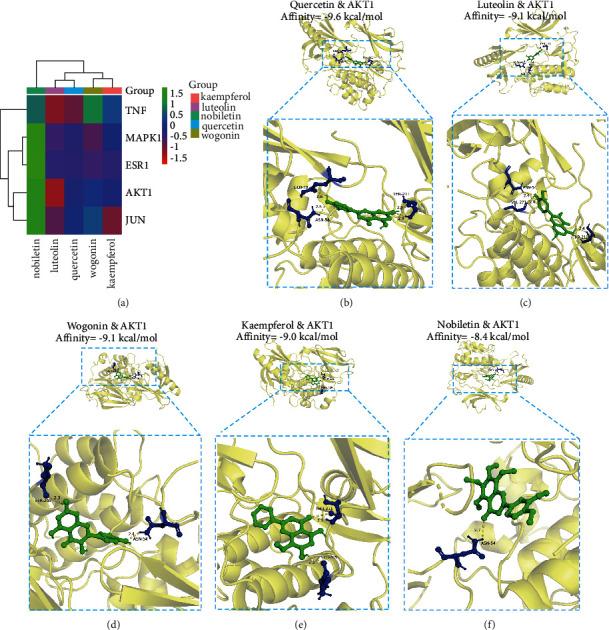
Docking analysis. (a) Heat map of the binding energy between the macromolecular protein and small molecule compounds of an affinity less than −5 kcal/mol. (b)–(f) The top five molecular docking binding affinities.

A total of 25 pairs were evaluated by the docking simulation. Molecular docking results showed that quercetin, kaempferol, luteolin, wogonin, and nobiletin could spontaneously bind to key targets in AKT1, JUN, MAPK1, ESR1, and TNF protein receptors. The binding energies less than −5 kcal/mol are shown in Figure 4(a). The top five docking binding energies were obtained for AKT1-quercetin docking (−9.6 kcal/mol), AKT1-luteolin docking (−9.1 kcal/mol), AKT1-wogonin docking (−9.1 kcal/mol), AKT1-kaempferol docking (−9.0 kcal/mol), and AKT1-nobiletin docking (−8.4 kcal/mol). However, quercetin bound to 6HHF (AKT1) via forming three hydrogen bonds with GLN-76, ASN-54, and THR-211. Luteolin bound to 6HHF (AKT1) via three hydrogen bonds to VAL-271, ASN-54, and THR-211. Wogonin bound to 6HHF (AKT1) via two hydrogen bonds with SER-205 and ASN-54. Kaempferol bound to 6HHF (AKT1) via two hydrogen bonds with SER-205 and THR-211. Kaempferol bound to 6HHF (AKT1) via one hydrogen bond with ASN-54 (Figures 4(b)–4(f)). It is generally accepted that a binding energy of <−7.0 kcal/mol indicates strong binding activity between the ligand and receptor. These results show that the effects of KJK on UC may be mediated by AKT1 via the active component group dominated by quercetin, kaempferol, luteolin, wogonin, and nobiletin. To verify these results, we used UPLC-QEMS technology and animal experiments.

### 3.5. Analysis of Kui Jie Kang Water Extract by UPLC-QE-MS

**Figure 5:**
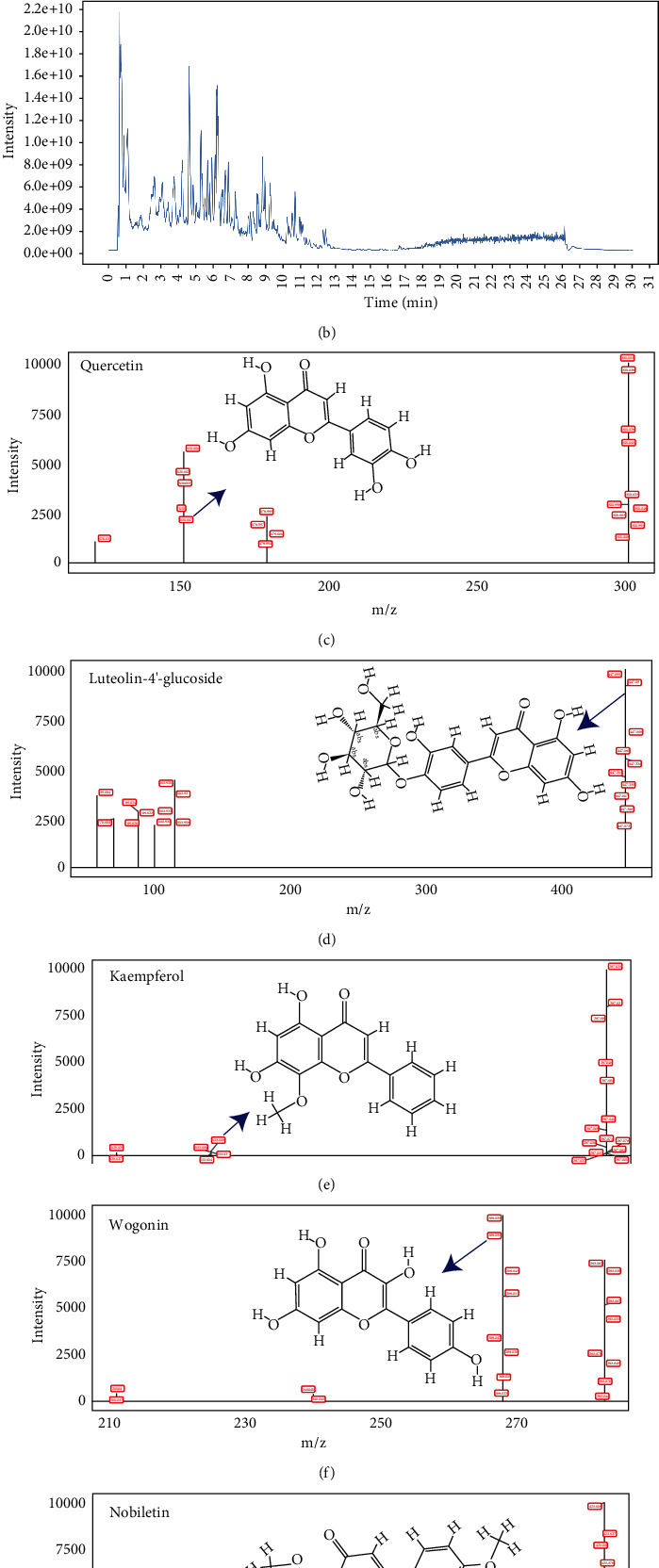
UHPLC-QE-MS chromatograms of KJK under positive and negative ion modes. (a) Positive ion mode; (b) negative ion mode. (c)–(g) Secondary mass spectra of quercetin, luteolin, kaempferol, wogonin, nobiletin, and the exact structure.

To verify the main active components of KJK obtained by network pharmacology, comprehensive analyses of the accurate relative molecular weight and MSn multi-stage mass spectrometry were performed. The total ion flow diagram of KJK in positive and negative ion modes is shown in Figure 5. Quercetin, kaempferol, luteolin, wogonin, and nobiletin in KJK were determined based on the accurate relative molecular weight, ionic fragments, and reference materials. These five potential biomarkers were detected in the KJK decoction.  Table 4 provides basic information on the main active components of KJK with therapeutic effects in UC.

### 3.6. Therapeutic Effect of Kui Jie Kang on Ulcerative Colitis

**Figure 6:**
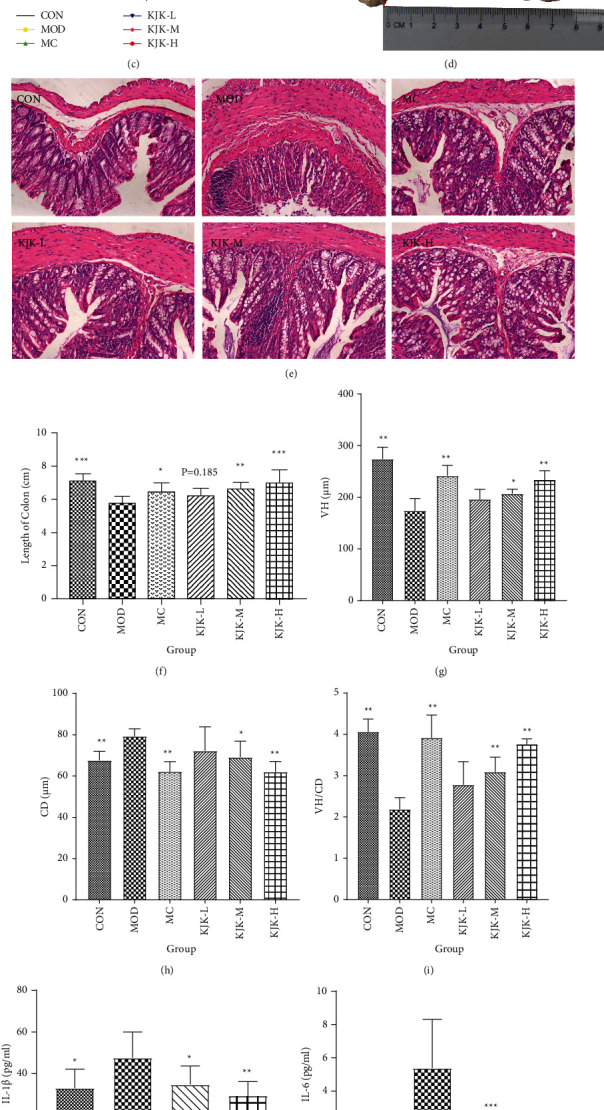
KJK ameliorates DSS-induced experimental UC. (a) Treatment regimen for all mice groups. (b) Body weight. (c) Changes in disease activity index (DAI) over time. (d) Colon length. (e) Representative images of hematoxylin and eosin stained sections (H & E staining, 200×). (f) Colon length statistics. (g)–(i) Effects of KJK on villi height (VH) and crypt depth (CD) in mice with UC. (j)–(l) Expression of IL-1*β*, IL-6, and TNF-*α* proteins in the colon tissues of mice in different treatment groups (*n* = 8). Measurement data are expressed as mean ± standard error of the mean. One-way analysis of variance (ANOVA) was conducted for the group comparison. ^*∗*^*P* < 0.05, ^*∗∗*^*P* < 0.01, ^*∗∗∗*^*P* < 0.001, ^*∗∗∗∗*^*P* < 0.0001 compared with the MOD group. ^#^*P* < 0.05, ^##^*P* < 0.01, ^###^*P* < 0.001 compared with the CON group.

Next, we evaluated the effect of KJK on mice with 2.5% DSS-induced UC (Figure 6(a)). Seven days after treatment with 2.5% DSS, mice in the CON group had a glossy hair color and were sensitively active. The mice in the MOD group exhibited weight loss, reduced diet intake and activity, huddling together behavior, bloody stools, irregular stools, and some had vertical hair or an arched back. The weight, colon length, and DAI score of mice with 2.5% DSS-induced colitis decreased significantly from day 1 to day 14 (*P* < 0.05; Figures 6(b)–6(d)). Meanwhile, the colonic glands of the CON group showed an orderly arrangement, the tissue structure was complete, and no histopathological changes, such as congestion, necrosis, or edema, were observed (Figures 6(e) and 6(f)). In the MOD group, colonic tissues showed severe intestinal epithelial injury, recess atrophy or loss, mucosal necrosis, disappearance of the original tissue structure in the necrotic area, cell edema, and substantial inflammatory cell infiltration (Figures 6(g)–6(l)).

KJK and mesalazine effectively reversed the significant changes in body weight and colon length in colitis mice; these parameters were significantly lower in the MC and KJK-L/M/H groups than in the MOD group (Figure 6(c)). The colon lengths in the KJK-L/M/H groups were significantly longer than that in the MOD group, especially in the KJK-H group (Figures 6(d) and 6(f)). Colonic epithelial injury and inflammatory cell infiltration were reduced in the mice in each treatment group, and crypt inflammation, tissue edema, and necrosis were improved. Only mild intestinal epithelial injury and a small amount of inflammatory cell infiltration were observed in the colonic tissue of mice in the KJK-H group (Figures 6(e), 6(g)–6(i)). The effect of KJK on the expression of inflammatory factors in the colon tissue of UC mice was further analyzed by ELISA. The expression levels of the proinflammatory cytokines IL-6 and IL-1*β* were significantly higher in the colon of MOD mice than in those of the CON mice (*P* < 0.05). However, compared to levels in the MOD group, TNF-*α*, IL-6, IL-1*β*, and TNF-*α* expression levels were significantly lower in the colons of the KJK-M and KJK-H groups (*P* < 0.01; Figures 6(j)–6(l)). These results indicate that KJK can alleviate colonic injury and the gross symptoms of DSS-induced colitis in mice.

### 3.7. Effect of Kui Jie Kang on PI3K and AKT Expression in the Colon of Mice with Ulcerative Colitis

**Figure 7:**
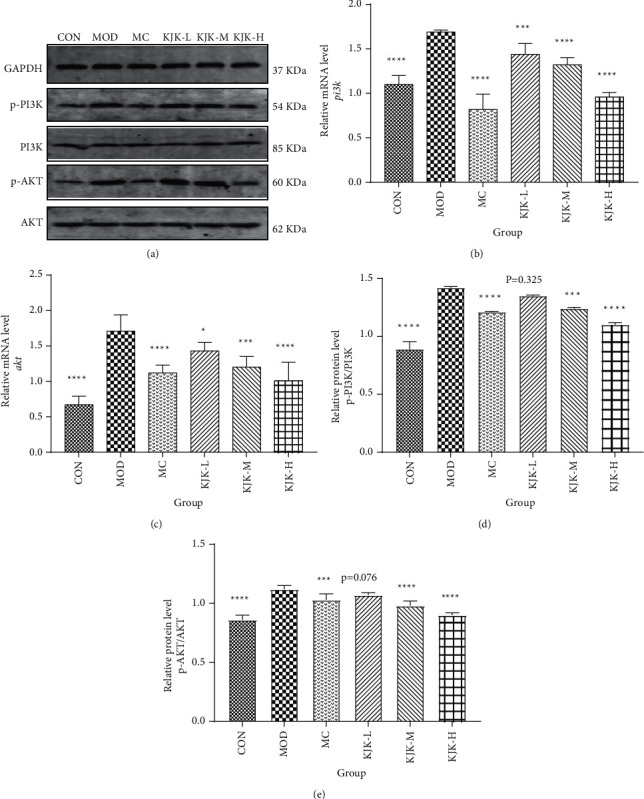
KJK inhibits the activation of PI3K/AKT in UC mice induced by 2.5% DSS. (a) Representative immunoblotting images of p-PI3K, PI3K, p-AKT, AKT, and GAPDH. (b) and (c) Effect of KJK on the mRNA expression of *pi3k* and *akt* in the colon of mice with UC. (d, e) Effect of KJK on p-PI3K/PI3K and p-AKT/ AKT protein expression in the colon of mice with UC. Data are expressed as mean ± SEM. *n* = 3. One-way ANOVA was conducted for the group comparison. ^*∗*^*P* < 0.05, ^*∗∗*^*P* < 0.01, ^*∗∗∗*^*P* < 0.001, ^*∗∗∗∗*^*P* < 0.0001 compared with the MOD group.

The bioinformatics analysis showed that AKT1 was the most important target, and the PI3K-AKT signaling pathway was the most important pathway for the treatment of UC. Therefore, to further elucidate the mechanism underlying the anti-UC activity of KJK, we verified the expression of proteins in the PI3K-AKT signaling pathway using RT-qPCR and Western blotting (Figure 7). Compared with those of the CON group, the relative mRNA levels of *pi3k* and *akt*, and the relative protein level of p-PI3K/PI3K and p-AKT/AKT were significantly higher in the MOD group (*P* < 0.0001).

The increases in PI3K and AKT in the MOD group were significantly attenuated in all KJK treatment groups, and relative *pi3k* mRNA expression in the KJK-L and KJK-M groups exceeded 1 (Figures 7(b) and 7(c)). As the drug dose increased, *pi3k*mRNA expression gradually decreased. In addition, compared with levels in the MOD group, the abundance of p-PI3K/PI3K and p-AKT/AKT was significantly lower in each treatment group (*P* < 0.05; Figures 7(d) and 7(e)). These results indicate that KJK exerts an anti-inflammatory effect by inhibiting the transcription and phosphorylation of proteins associated with the PI3K-AKT signaling pathway. Overall, these findings further support the notion that the therapeutic effect of KJK in UC occurs via the PI3K-AKT signaling pathway.

## 4. Discussion

UC in TCM belongs to the categories “dysentery,” “intestinal wind,” “intestinal bleeding,” and “great diarrhea” [19]. According to the theory of TCM, the principle of “Jun, Chen, Zuo, and Shi” (which translates to monarch, minister, adjuvant, and guiding medicine) has a long history in the theory of Chinese medicine treatment. The “Jun” medicine in KJK is *Atractylodes macrocephala*. The “Chen” medicine in KJK is *Poria cocos*, *Paeoniae radix alba*, *Saposhnikovia divaricata*, *Platycodon grandiflorus*, Citri Reticulatae Pericarpium. The “Zuo” medicine in KJK is *Codonopsis pilosula*, *Bletilla striata*, *Sanguisorba officinalis*, *Panax notoginseng*, *Patrinia villosa*, *Dioscorea rhizoma*. The “Zuo” medicine in KJK is *Glycyrrhiza uralensis*. The whole medical compound has the functions of soothing the liver, strengthening the spleen, clearing heat, cooling blood, and stopping bleeding. However, each TCM has thousands of components, and its multi-component, multi-channel, and multi-target nature make it difficult to evaluate the treatment effects and underlying mechanism in a scientific framework.

The development of network pharmacology has provided new research ideas and technical means for studying diseases and the mechanisms underlying the effects of TCM compounds [20]. Based on multi-component and multi-target action research concepts, we collected 145 active components and 412 targets from multiple databases using a network pharmacological approach. Bioinformatics analysis further revealed 205 genes closely related to the pharmacodynamic activity of KJK. Moreover, active ingredient-target network analysis for KJK was identified including quercetin, kaempferol, luteolin, wogonin, and nobiletin, flavonoids as the top 10 key pharmacodynamic molecules. Meanwhile, we evaluated the KJK decoction by UPLC-QE-MS to verify that it contains quercetin (*m*/*z* 301.034853), kaempferol (*m*/*z* 287.0544552), luteolin-4-O-glucoside (*m*/*z* 447.0960827), wogonin (*m*/*z* 283.0606575), and nobiletin (*m*/*z* 425.119079).

Quercetin is a polyphenolic flavonoid that functions as an antioxidant, radical scavenger, protein kinase inhibitor, as well as an anti-inflammatory, antiviral, and antibacterial agent. As such, it has been used to treat or prevent diverse conditions, including hypercholesterolemia, rheumatic diseases, and infections [21–23]. Quercetin can reduce mRNA levels of TNF-*α*, lipocalin-2, and other genes, regulate the structure of the intestinal flora, and inhibit the expression of proinflammatory factors in UC [24], which further demonstrates the importance of inflammatory responses in UC pathology [8]. Kaempferol is a tetrahydroxyflavone that functions as an antioxidant by reducing oxidative stress, and has strong anti-inflammatory pharmacological activity [25]. Indeed, previous animal studies have shown that oral administration of kaempferol can protect colonic mucosa from DSS-induced UC by alleviating the inflammatory response [26]. Luteolin/luteolin-4-O-glucoside is also a naturally occurring flavonoid, with potential antioxidant, anti-inflammatory, apoptosis-inducing, and nerve protective effects. Moreover, it has anti-inflammatory and antioxidant effects though modulation of the NF-*κ*B pathway [27, 28]. The main reason is that flavonoids are widely distributed in Chinese herbal medicine and natural products, and have anti-inflammatory, antibacterial, and antioxidant activities [29, 30].

Based on the above research results, we constructed a PPI network, which facilitated the indentation of 15 potential core targets. Combined with the molecular docking results, AKT1, JUN, MAPK1, ESR1, and TNF were ultimately characterized as key targets of KJK in the treatment of UC. However, AKT is at the core of the key target network relationship of KJK in the treatment of UC. Interestingly, the main active components quercetin, kaempferol, luteolin, wogonin, and nobiletin of KJK have good binding activity with AKT1. AKT1 is one of three closely related serine/threonine-protein kinases (AKT1, AKT2, and AKT3) called the AKT kinase and is pivotal as a downstream effector of phosphatidylinositol kinase PI3K [31]. Moreover, abnormal activation of the PI3K pathway plays an important role in the pathogenesis of UC [32]. PI3K is a family of intracellular conducting enzymes [33]; when all sites are phosphorylated, AKT on the cell membrane becomes fully activated and is released from the cell membrane into the cytoplasm or nucleus for signal transmission to perform its biological functions. Therefore, p-AKT can be used as an index to judge the activity of PI3K [34].

The KEGG pathway enrichment results showed that KJK treatment of UC primarily involved the PI3K-AKT, cAMP, MAPK, calcium, Ras, TNF, Foxo, and toll-like receptor signaling pathways. Furthermore, the PI3K/AKT signaling pathway was the most enriched. In recent years, the role of this pathway in intestinal inflammation and tumors has garnered increasing attention [35]. In fact, the PI3K/AKT pathway is implicated in the regulation and release of proinflammatory cytokines, which in turn participates in the development of UC, with PI3K/AKT abundance augmented in mice with UC [36]. More specifically, PI3K/AKT contribute to significantly increased expression of IL-6, IL-1*β*, and TNF-*α* [37–39]. Meanwhile, application of a PI3K/AKT inhibitor has been shown to improve various symptoms of UC in mice, including reduced growth, loose stools, and colonic bleeding, while further repressing activation of the PI3K/AKT pathway [35, 36].

In line with the above findings, we found that KJK could effectively reverse the significant changes in body weight, colon weight index, colon weight, and colon length in mice with colitis. Moreover, colonic epithelial injury and inflammatory cell infiltration were reduced by treatment with KJK resulting in improved crypt inflammation, tissue edema, and necrosis. At the same time, KJK was found to significantly reduce TNF-*α*, IL-6, and IL-1*β* abundance in UC mice colons and downregulate the expression of *pi3k* and *akt*, as well as the protein abundance of p-PI3K/PI3K and p-AKT/AKT. In summary, KJK can reduce the expression of genes in the PI3K/AKT signaling pathway and inhibit the activation of PI3K/AKT signaling in the colon, thus alleviating DSS-induced UC in mice.

Certain limitations were noted in the current study. First, it is necessary to verify which relevant genes are enriched in the PI3K/AKT signaling pathway using *in vitro* analyses. Moreover, a rescue experiment will serve to further clarify the mediating effect of KJK and its key components on the PI3K/AKT pathway in the prevention and treatment of UC. Second, it is necessary to carry out more in-depth experimental research and verification from different dimensions in combination with the results of molecular docking and pathway enrichment analysis to further reveal the modern scientific connotation of “dysentery” TCM in the prevention and treatment of UC.

## 5. Conclusion

The aim of this study was to explore the molecular mechanisms underlying the therapeutic effect of KJK against UC via a comprehensive bioinformatics analysis. More specifically, we aimed to identify the main active components, key targets, and associated pathways involved in treatment of UC with KJK. Collectively, the results show that KJK primarily elicits its therapeutic effect on UC via modulation of the PI3K/AKT signaling pathway. Hence, KJK may represent a valuable therapeutic agent for the treatment of patients with UC. Nevertheless, additional molecular experiments are needed for further validation of the findings described herein.

## Figures and Tables

**Table 1:** Disease activity index (DAI) scores.

Parameter	Scoring criteria
Body weight loss	0 points = none, 1 point = 1–5% loss, 2 points = 5–10% loss
Diarrhea	0 points = normal, 2 points = loose stools, 4 points = watery diarrhea
Fecal bleeding	0 points = no bleeding, 2 points = slight bleeding, 4 points = gross bleeding

DAI = (body weight loss + diarrhea + fecal bleeding)/3.

**Table 2:** Topological parameters of target nodes for KJK treatment of UC.

name	DC	BC	CC	LAC
STAT3	77	0.045274	0.58409786	24.28571429
TP53	77	0.10804017	0.58950617	19.27272727
AKT1	72	0.05648135	0.57357357	20.66666667
TNF	69	0.04957359	0.56508876	20.23188406
IL6	67	0.03643073	0.55847953	21.25373134
MAPK3	63	0.04141007	0.56176471	20.38095238
JUN	58	0.022095	0.55202312	21.68965517
EGFR	58	0.04308535	0.54885057	20.4137931
HSP90AA1	57	0.02596477	0.53501401	17.89473684
VEGFA	54	0.01922315	0.53954802	20.59259259
IL1B	54	0.02528887	0.52908587	18.33333333
MAPK1	52	0.02487655	0.53501401	16.88461538
CASP3	49	0.01588638	0.51761518	18.16326531
MYC	46	0.01799492	0.51069519	18.91304348
ESR1	45	0.03593579	0.52043597	16.97777778

**Table 3:** Molecular docking of the main active ingredients of KJK and core proteins.

MOL ID	Core active ingredient	Binding energy/kcal.mol^−1^				
		AKT1	JUN	ESR1	AKTI	TNF
MOL000098	Quercetin	−9.56	−8.00	−7.97	−7.71	−4.82
MOL000006	Luteolin	−9.11	−7.67	−7.83	−7.62	−4.80
MOL000422	Kaempferol	−8.98	−7.49	−8.57	−7.79	−4.59
MOL000173	Wogonin	−9.08	−8.09	−7.96	−7.51	−4.65
MOL005828	Nobiletin	−8.44	−7.08	0.73	−6.71	−4.62

**Table 4:** Detection of the main active components of the KJK decoction in the treatment of UC.

MOL ID/(compound)	tR/min	Formula	Adduct	*m*/*z*	Mass error (ppm)	MS/MS	Intensity	References
MOL000098 (quercetin)	7.08	C_15_H_10_O_7_	[*M* − *H*]^−^	301.0349	0.48	151.004	1234910	[14]
MOL000006 (luteolin/luteolin-4′-O-glucoside)	7.36	C_21_H_20_O_11_	[*M* − *H*]^−^	447.0961	0.19	447.099	871413	[15]
MOL000422 (kaempferol)	5.37	C_15_H_10_O_6_	[*M* + *H*]^+^	287.0545	1.59	153.018	72766	[16]
MOL000173 (wogonin)	7.15	C_16_H_12_O_5_	[*M* − *H*]^−^	283.0607	1.21	268.039	688743	[17]
MOL005828 (nobiletin)	9.8	C_21_H_22_O_8_	[*M* + *H*]^+^	425.1191	0.19	395.064	282093	[18]

## Data Availability

The datasets used and/or analyzed during the current study are available from the corresponding author on reasonable request.
